# Familiarity in Rural Life: Protocol for a Scoping Review and Concept Analysis

**DOI:** 10.2196/36930

**Published:** 2022-06-22

**Authors:** Marilyn A Swan, Luke J Gietzen, Barbara B Hobbs

**Affiliations:** 1 School of Nursing College of Allied Health & Nursing Minnesota State University, Mankato Mankato, MN United States; 2 College of Social & Behavioral Sciences Minnesota State University, Mankato Mankato, MN United States; 3 College of Nursing South Dakota State University Rapid City, SD United States

**Keywords:** familiarity, scoping review, rural, nursing, nurse, healthcare professional, health care professional, healthcare worker, health care worker

## Abstract

**Background:**

Familiarity is a concept often used in literature but is not well defined or understood. As a key concept in rural nursing theory, the conceptual understanding of familiarity is currently incomplete. The findings from this scoping review will inform a concept analysis using Walker and Avant’s method and to identify and define the missing key components of familiarity.

**Objective:**

The objective of this scoping review is to examine and analyze what is known in the existing literature about the concept of familiarity.

**Methods:**

The Joanna Briggs Institute scoping review framework guided the identification of literature published from 2016 to 2022 on familiarity. Following the PRISMA-ScR (Preferred Reporting Items for Systematic reviews and Meta-Analyses extension for Scoping Reviews) reporting standard, the familiarity scoping review is registered on Open Science Framework (registration digital object identifier: 10.17605/OSF.IO/ZB8VF). A total of 8 databases, including PubMed, CINAHL (Cumulative Index to Nursing and Allied Health Literature) Plus with full text, APA PsychInfo, Communication Source, EBSCO MegaFILE, Medline, Nursing & Allied Health Database, and ScienceDirect, will be searched for 22 search terms. Covidence software will be used to manage the scoping review with each citation independently reviewed by 2 research team members for eligibility. Eligibility will be determined using a 2-level process. Each title and abstract will be screened for eligibility; for citations deemed eligible, a full-text article review will be conducted. The scoping review is expected to locate a large body of literature, and eligibility criteria will be refined during the title and abstract screening process. In addition, reference list scanning will be performed to locate relevant literature.

**Results:**

Familiarity data will be collected beginning October 2021 with anticipated completion in March 2022. Dissemination of findings will occur through scholarly presentations and in rural-focused and nursing publications in 2022 or 2023. The findings from this review will further the understanding of familiarity and how it affects rural life and nursing practice.

**Conclusions:**

This review will support a full understanding and add clarity to the concept of familiarity as a component of rural life. These new insights will advance the understanding of how familiarity influences rural health care practice. The concept analysis will provide theoretical support for rural nursing theory and promote an understanding of the interrelationships of rural concepts.

**International Registered Report Identifier (IRRID):**

PRR1-10.2196/36930

## Introduction

Familiarity is a common word used in everyday communication and in all settings. Most often, familiarity denotes knowledge of a person, place, or thing, or is indicative of informal behavior [1]. As people go about life, familiar interactions with or exposures to objects, people, and locations are stored as memories in the brain [2]. Thus, people become accustomed to what is repeatedly experienced. This is particularly true for people who live in rural or small locations. Rural dwellers often report having a strong experience with familiarity, particularly with people and places [3,4]. Small locations, with limited numbers of individuals, promote increased knowledge and greater familiarity [5]. As such, it makes sense that early rural nurse researchers identified familiarity as a concept present in rural locations [4,6]. More specifically, familiarity is identified as a core concept in rural nursing theory [7,8].

Williams et al [9] identified that rural nursing research is hampered by a lack of understanding of rural concepts. Many rural concept analyses are dated, completed in 1998 or before, and lack rigorous literature reviews using current guidelines and methods. There is a need to develop rural concepts so a strong theoretical foundation is established, which will guide future research, particularly on rural topics [9].

Within the rural nursing literature, the concept of familiarity is not well defined or understood. The original concept analysis was incomplete; a definition of familiarity was presented and defining attributes, or characteristics, were identified [7]. As specified in Walker and Avant’s [10] concept analysis process, antecedents, consequences, and empirical referents were not identified [8]. In addition, an unknown number of articles were reviewed, and limited disciplines were included in the analysis [7].

Our interest in familiarity grew from a completed concept analysis on the rural concept *lack of anonymity* [11]. In that analysis, familiarity was identified as a consequence of lack of anonymity [11]. As our theoretical work developed, we recognize that familiarity requires further exploration. The process was arduous, as familiarity is commonly used as a word to replace descriptive words such as knowledge, experience, awareness, and others. Thus, literature searches for familiarity yielded extensive amounts of literature, from multiple disciplines, without a thorough understanding of the concept. After two limited attempts to examine familiarity as a concept [12,13], it became apparent that a scoping review was necessary to fully explore the concept.

Understanding familiarity as a concept is foundational to rural nursing theory and to the influence of familiarity on everyday life. A scoping review of literature will be conducted on familiarity. Findings from this scoping review will inform a concept analysis using Walker and Avant’s [10] process to understand key components of familiarity and how familiarity relates to rural nursing theory and practice. The aim of this review is to examine and analyze what is known in the existing literature about the concept of familiarity.

## Methods

### Scoping Review

A scoping review supports clarification of the concept and the exploratory nature of the review [14]. Walker and Avant [10] support a broad, multidisciplinary review of the literature to gain a full understanding of a concept. The Joanna Briggs Institute scoping review methodology, as outlined by Aromataris and Munn [15], builds on the seminal work of Arksey and O’Malley [16] and will be the framework for this review. In accordance with the PRISMA-ScR (Preferred Reporting Items for Systematic reviews and Meta-Analyses extension for Scoping Reviews) reporting standard [17], the familiarity scoping review is registered on Open Science Framework (registration digital object identifier: 10.17605/OSF.IO/ZB8VF).

#### Step 1: Identify the Purpose

The research question guides the review, and for a concept analysis, it must be broad and comprehensive [10,15]. The research question developed by the research team is “What is known from the existing literature about the concept of familiarity?”

#### Step 2: Search Strategy

For the review, 8 databases will be searched for relevant literature, including PubMed, CINAHL (Cumulative Index to Nursing and Allied Health Literature) Plus with full text, APA PsychInfo, Communication Source, EBSCO MegaFILE, Medline, Nursing & Allied Health Database, and ScienceDirect. Gray literature is not included in the review; however, reference list scanning will be completed during the literature screening procedure. Thus, literature outside of the search dates may be included if deemed relevant.

A challenge for this review is the pervasive use of the word and quantity of literature that may be located during the search. As suggested by Aromataris and Munn [15], the search terms must be both specific and broad to access relevant literature for full conceptualization while balancing the need for a manageable amount of literature for a review. The search parameters include the word “familiarity” in the abstract or title and from a peer-reviewed source from January 1, 2016, to 2022 (Textbox 1). The review was conducted late in 2021, and 2022 literature may be released and will be included in the scoping review. The search terms include “empathy and familiarity” OR “memory and familiarity” OR “recognition and familiarity” OR cognit* and familiarity” OR “spatial familiarity” OR “contextual familiarity” OR “personal familiarity” OR “belonging familiarity” OR “relationship and familiarity” OR “lack of anonymity and familiarity” OR “privacy and familiarity” OR “confidentiality and familiarity” OR “nurs* and familiarity” OR “medicine and familiarity” OR “social work and familiarity” OR “healthcare and familiarity” OR “rural and familiarity” OR “gaming and familiarity” OR “social media and familiarity” OR “culture and familiarity” OR “religion and familiarity” OR “spirit* and familiarity.” The search terms will be run in each of the selected databases using the asterisk symbol as an end-of-root-word-truncation mark. The identified literature will be imported to the Covidence software program with duplicate records removed.

**Inclusion and exclusion criteria.**

**Inclusion criteria:**

- Define or directly discuss familiarity as a concept
- Apply to humanity or human beings
- Indicate a study result or finding on familiarity
- English language literature including international sources

**Exclusion criteria:**

- Studies or articles referring to animals or nonhumans
- Studies on genetics (human and nonhuman)

#### Step 3: Selection Process

The literature screening is composed of 2 separate reviews. Covidence software will be used to manage the scoping review with each citation independently reviewed by 2 research team members for eligibility. Eligibility will be determined using a 2-level process. Each title and abstract will be screened for eligibility; for citations deemed eligible, a full-text article review will be conducted. The scoping review is expected to locate a large body of literature, and eligibility criteria will be refined during the title and abstract screening process. In addition, reference list scanning will be used to locate relevant literature. The literature screening process is consistent with the screening strategy described by Aromataris and Munn [15].

The research team will have regular meetings (every 1-2 weeks) to discuss the project, refine eligibility criteria, and resolve conflicts on article eligibility. Resolution of disagreement on the eligibility of an article will occur during the full team meeting. The majority will decide eligibility. If the team is unable to establish consensus, the project leader will resolve the disagreement.

##### Title and Abstract Screening

Prior to the start of literature screening, research team members will receive education from the project leader followed by a pilot test. The purpose of the education and pilot study is to promote consistency in evaluating literature among the team members. At a team meeting, each team member will receive detailed instructions on how to use the Covidence software. Additionally, the inclusion and exclusion criteria will be discussed in detail and questions answered. Following the instruction, a pilot test will be conducted. For the pilot test, each pair of researchers will have 25 randomly selected abstracts to review based on the inclusion and exclusion criteria, followed by an interrater reliability (IRR) calculation between each pair of team members. The expected IRR achievement between each pair of team members is >0.75 [15]. Upon conclusion of the pilot study, the team will meet to discuss discrepancies and will consider modifications to the inclusion and exclusion criteria. Consistent with Arksey and O’Malley [16], modifications to the inclusion and exclusion criteria can be made as the team understands the scope of the literature being reviewed.

##### Full Text Screening for Eligibility

Prior to the start of the full text screening, the full-text articles will be uploaded in the Covidence software, and the research team will meet to discuss the screening process, including the use of the Covidence software at this phase of the review. Once completed, each team member will begin to review articles, with an IRR calculated when each team pair reviews at least 25 articles. The expected IRR achievement between each pair of team members is >0.75; if not met, the research team will meet for additional instruction and discussion.

Reference list scanning will be carried out during the full text screening process. Research team members will send citations to the project leader. Upon conclusion of the full text screening, the project leader will present the reference list articles for consideration to the research team. The majority will decide eligibility. If the team is unable to establish consensus, the project leader will resolve the disagreement.

#### Step 4: Charting the Data

The research team has developed a draft charting table for data extraction (Textbox 2). Data will be charted using a descriptive method that aligns to the scoping review’s research question and extract descriptions, definitions, and other relevant findings on familiarity. Key to this review is to identify all the ways familiarity is used, both implicit and explicit, in the identified literature.

Aromataris and Munn [15] suggest that the draft charting table be piloted by each team member. For this review, each team member will trial the charting table by extracting data from 3 articles [15]. At the end of the trial, the research team will meet to determine the adequacy of the charting table and make modifications, if needed. Owing to the broad nature of the concept, the data charting process is considered iterative [15]. Thus, the charting table may be continually updated by the team, if deemed necessary, to fully understand the concept.

Similar to the literature screening process, selected articles will have data charted by 2 team members. Team members will independently extract data from the selected literature. In the event of disagreement on the data extracted, the research team will discuss at a full team meeting. The majority will decide data disagreements. If the team is unable to come to an agreement, the project leader will resolve the disagreement.

**The data and details for extraction.**

- Article information:
  Authors
  Year of publication
  Title
  Type of study
  Discipline
  Country of origin
- Study setting
- Study population
- Types of data sources
- Was familiarity measured or described? (Yes or no)
- Familiarity definition or description
- Relevant findings

#### Step 5: Data Analysis

The data analysis will be guided by Walker and Avant’s [10] 8-step concept analysis process, starting with step 4:

1. Select a concept.
2. Determine the aims or purpose of analysis.
3. Identify all uses of the concept that you can discover.
4. Determine the defining attributes.
5. Identify a model case.
6. Identify borderline, related, contrary, invented, and illegitimate cases.
7. Identify antecedents and consequences.
8. Define empirical referents.

Central to the process is to identify the defining attributes of familiarity, which reveal the characteristics of the concept [10]. For the analysis, each team member will independently review the data tables and begin to cluster data that offer broad insight into the concept of familiarity. The process is iterative as the team identifies phenomena and occurrences in the data. The defining attributes will be considered complete when each attribute can “stand alone” and fully capture the essence of the concept [10].

Identification of *cases* demonstrates what the concept is and what it is not in situational life examples. A model case will demonstrate familiarity using the defining attributes identified [10]. To delineate related concepts that may be close to familiarity, additional cases will be developed, including borderline, related, contrary, invented, and illegitimate cases [10].

Once clarity on the defining attributes is achieved, the antecedents and consequences will be identified. Antecedents are “those events or incidents that must occur or be in place prior to the occurrence of the concept” [10]; consequences are “those events or incidents that occur as a result of the occurrence of the concept—in other words, the outcomes of the concept” [10]. This portion of the analysis supports a theoretical understanding of how the concepts fit together.

The final part of the analysis is to identify the empirical referents or how familiarity is revealed in everyday life [10]. The empirical referents represent categories that will support the development of a measure [10].

## Results

The scoping review will begin in October 2021 and is expected to be completed in March 2022. A flow diagram will be developed to demonstrate how literature screening will be managed during the selection process [17]. Dissemination of the results of the scoping review and concept analysis will be carried out through peer-reviewed scholarly presentation at a research conference in March 2022 and in a rural nursing publication in 2022 or 2023. Additional manuscripts are planned for publications with a rural focus and readership.

The findings from the review and analysis are foundational to support a common understanding of the concept of familiarity that supports future research, theory development, and health care initiatives [10].

## Discussion

In rural practice settings, familiarity is a recognized concern for nurses and health care professionals. Nurses in rural practice experience familiarity with patients, families, and the environment without recognizing it as a factor in rural settings. Research into rural issues is complicated by a general lack of understanding of basic concepts such as familiarity, which exist in rural life.

The outcomes of this scoping review and concept analysis will fully conceptualize familiarity as a component of rural life and may support the development of a guide on how familiarity may affect nurses and health care professionals in rural practice locations. In addition, the concept analysis will provide theoretical support for rural nursing theory and extend the model of lack of anonymity by understanding the interrelationship among rural concepts [13].

## Abbreviations

APA: American Psychological Association
CINAHL: cumulative index to nursing and allied health literature
DOI: digital object identifier
IRR: interrater reliability
PRISMA-ScR: Preferred Reporting Items for Systematic reviews and Meta-Analyses extension for Scoping Reviews
